# Effect of Socioeconomic Status on Cervical Cancer Screening Behaviour Among Mothers of Adolescent Girls in Lagos, Nigeria: A Secondary Analysis of the *mHealth-HPVac* Study

**DOI:** 10.21203/rs.3.rs-8126074/v1

**Published:** 2026-01-09

**Authors:** Kehinde S. OKUNADE, Adaiah SOIBI-HARRY, Ayomide I. FAYINTO, Hameed ADELABU, Iyabo Y. ADEMUYIWA, Ayokunle M. OLUMODEJI, Adetunji O. ADENEKAN, Muisi A. ADENEKAN, Festus O. OLOWOSELU, Temitope V. ADEKANYE, Olufemi THOMAS-OGODO, Oziegbe OGHIDE, Yusuf A. OSHODI, Babasola O. OKUSANYA

**Affiliations:** 1. Department of Obstetrics & Gynaecology, College of Medicine, University of Lagos, Idi-Araba, Lagos, Nigeria; 2. Department of Obstetrics & Gynaecology, Lagos University Teaching Hospital, Idi-Araba, Lagos, Nigeria; 3. Centre for Clinical Trials, Research and Implementation Science, College of Medicine, University of Lagos, Idi-Araba, Lagos, Nigeria; 4. Obstetrics & Gynaecology, Lagos Island Maternity Hospital, Lagos, Nigeria; 5. Department of Nursing Science, College of Medicine, University of Lagos, Idi-Araba, Lagos, Nigeria; 6. Department of Ophthalmology, College of Medicine, University of Lagos, Idi-Araba, Lagos, Nigeria; 7. Department of Haematology and Blood Transfusion, College of Medicine, University of Lagos, Idi-Araba, Lagos, Nigeria; 8. Obstetrics & Gynaecology, Lagos State University Teaching Hospital, Ikeja, Lagos, Nigeria; 9. Department of Emergency Medicine, Lagos University Teaching Hospital, Idi-Araba, Lagos, Nigeria

**Keywords:** Cervical cancer, screening uptake, socioeconomic status, education, Nigeria

## Abstract

**Background:**

Cervical cancer remains a preventable malignancy with high morbidity and mortality in low- and middle-income countries (LMICs). Screening uptake is suboptimal in sub-Saharan Africa, and the influence of socioeconomic status (SES) on screening behaviour among women remains insufficiently explored.

**Aim:**

To determine the effect of SES on cervical cancer screening uptake among mothers of vaccine-eligible adolescent girls in Lagos, Nigeria.

**Methods:**

This secondary analysis used baseline data from the *mHealth-HPVac* trial, including 180 sexually active mothers of unvaccinated girls aged 9–14 years. The primary outcome was self-reported cervical cancer screening within the previous 10 years. A multivariable logistic regression model was used to examine the association between SES and screening uptake, adjusting for age and tribe. Education and income were tested as an interaction term but excluded due to model instability.

**Results:**

Overall, 51.7% of participants reported prior cervical screening. Women who had been screened were significantly older than unscreened women (45.4±6.7 vs 41.7±7.2 years; p<0.001). Screening uptake did not differ significantly by marital status, education, employment, income class, tribe, or residential distance to screening facilities. In adjusted models, neither middle-income (adjusted odds ratio=1.72, 95% CI: 0.75–3.98) nor high-income status (adjusted odds ratio=1.22, 95% CI: 0.45–3.30) was associated with screening.

**Conclusion:**

Cervical cancer screening uptake among mothers in Lagos is moderate, and SES was not independently associated with screening. Interventions should target structural or behavioural factors rather than focusing solely on economic disparities. Larger, adequately powered population-based studies are therefore needed to validate these findings and better delineate the socioeconomic gradients in screening behaviour among Nigerian women.

## Introduction

Cervical cancer remains one of the most consequential yet preventable malignancies affecting women globally [1]. Recent estimates from GLOBOCAN 2020 place it as the fourth most common cancer among women worldwide, accounting for over 604,000 new cases and 342,000 deaths annually [1]. The burden is profoundly inequitable, with approximately 90% of global cervical cancer mortality occurring in LMICs, driven largely by gaps in prevention, screening, and early detection [2]. Sub-Saharan Africa (SSA) bears the highest age-standardised incidence and mortality rates globally, with an incidence of 40.1 per 100,000 women—nearly seven times that of high-income regions [3]. Nigeria contributes significantly to this burden, recording an estimated 15,000 new cases annually and over 8,000 deaths, making cervical cancer the second leading cause of female cancer mortality in the country [4]. The socioeconomic consequences of cervical cancer are profound, with women in their productive years often bearing the burden, which ripples through families, workplaces and communities.

Evidence demonstrates that early detection through cytology, HPV testing, or visual inspection substantially reduces cervical cancer incidence and mortality [3]. Yet, screening uptake in SSA remains critically low, often below 25% [5,6]. Socioeconomic status (SES) is a well-established determinant of screening behaviour globally. Higher education and income levels have consistently been associated with improved health literacy, autonomous decision-making, and preventive health practices, including cervical screening [7,8]. However, emerging evidence from LMICs suggests that this relationship may be more complex, with contextual cultural, structural, and gender-related factors mediating how SES influences screening behaviour [9]. Particularly among mothers, key actors in family health-seeking behaviours, the role of SES in shaping cervical screening decision-making remains insufficiently explored.

This secondary analysis of the *mHealth-HPVac* study, an HPV vaccination intervention trial among mothers of unvaccinated adolescent girls in Lagos, Nigeria [10,11], offers a unique opportunity to leverage high-quality baseline data collected from a diverse urban population of women to provide a timely and rigorous examination of SES-related disparities in cervical cancer screening behaviour. By modelling socioeconomic indicators while adjusting for key demographic and access variables, the study aims to clarify whether SES independently predicts screening uptake in this population. Therefore, the objective of this study is to determine the effect of SES on cervical cancer screening behaviour among mothers of vaccine-eligible adolescent girls in Lagos, Nigeria. The central hypothesis is that higher SES, measured through a composite SES index comprising education and occupation, will be positively associated with cervical screening uptake, independent of access-related factors.

## Participants and Methods

### Study Design and Settings

This study is a descriptive cross-sectional design using the baseline data obtained from the *“mHealth-HPVac”* trial [10,11], a recently completed randomised, parallel-arm controlled study conducted from June 2024 to March 2025. The parent trial enrolled mothers of unvaccinated girls aged 9–14 years attending routine care at the General Outpatient (GOP) Clinics of the Lagos University Teaching Hospital (LUTH), Lagos, Nigeria. LUTH, the main teaching hospital of the College of Medicine, University of Lagos, is situated in an urban, socioeconomically diverse Lagos metropolis, providing a suitable environment for examining how socioeconomic gradients influence cervical cancer screening behaviour.

### Study population

For this secondary analysis, we included the complete baseline dataset of 180 sexually active women from the primary study [11]. Eligibility for inclusion required women to have full sociodemographic, socioeconomic, and cervical cancer screening history data. Women with a previous diagnosis of cervical cancer, those of non-Nigerian nationality, or individuals unable to provide reliable responses due to cognitive impairment were excluded.

### Study endpoints/outcomes

The primary outcome was the association between socioeconomic status (SES) and screening uptake, and the secondary outcome was the modifying effect of formal educational attainment on the association between income status and screening behaviour. Cervical cancer screening uptake was defined as self-reported receipt of at least one Pap test or HPV-based screening within the past 10 years, with verification through clinic records where available. The income class was derived using a transparent and replicable method informed by the Demographic and Health Survey (DHS) wealth index framework, which combines educational attainment and occupational level [12]. Educational attainment was categorised as None/Primary, Secondary, or Tertiary. Employment status was categorized as Unskilled, Semi-skilled, or Skilled/Professional. Variables extracted for analysis included age, parity, tribe, marital status, educational attainment, employment status, residential distance to the nearest screening facility, cervical cancer awareness, and screening uptake.

### Sample size calculation

As this study was a secondary analysis of baseline data from the *mHealth-HPVac* trial [11], the sample size was fixed at 180 participants. To assess the adequacy of this sample for detecting the association between SES and cervical cancer screening uptake, we performed post-hoc sample size calculation using standard formulas for comparing two proportions [13] with 80% power and a two-sided alpha of 5%. Under plausible assumptions for cervical cancer screening, with a prevalence of 40–50%, this sample size provides adequate power to detect approximately a 20-percentage-point difference in screening uptake. We also evaluated the adequacy of the sample for multivariable logistic regression using the events-per-variable (EPV) rule. With 93 women (51.7%) reporting prior screening (events), the dataset supports the inclusion of approximately 6–8 predictor variables at EPV≈10, indicating that the planned adjusted model is statistically appropriate.

### Participants Sampling and Study Procedure

We collected data in the primary study [11] through interviewer-administered electronic case report forms (eCRFs) on REDCap, hosted on secure institutional servers. The eCRF, previously published elsewhere as Supplementary Material 3 [10], captured sociodemographic characteristics, economic indicators (education and occupation), reproductive history, and cervical cancer awareness and screening behaviour. Residential distance was calculated using participants’ addresses geocoded to the nearest health facility offering screening. Quality control measures included daily supervision of data collectors, range and logic checks in REDCap, and weekly data audits. Identifiable information was stored separately from research data using unique study codes to preserve confidentiality in line with ICH-GCP (E6[R2]) guidelines.

### Data Analysis

Data analysis was conducted in Stata version 18 (StataCorp LLC, Texas, USA). Missing values were addressed using multiple imputation by chained equations under the assumption of missing at random. Descriptive statistics were used to summarize participants’ characteristics using means and standard deviation for normally distributed variables and medians with interquartile ranges for skewed variables. The association between SES indicators and screening uptake was examined using multivariable logistic regression. Significant variables (*p*<0.2) in the univariable model (age and tribe) were included as covariates to adjust for potential confounding. Model diagnostics, including multicollinearity assessment, goodness-of-fit tests, and residual analysis, were performed to ensure appropriate model specification. To examine whether educational attainment modified the effect of income on screening behaviour, an education × income interaction term was tested. However, inclusion of this interaction resulted in numerical instability, characterized by inflated variance inflation factors (VIF>10), wide confidence intervals, and poor model convergence. For this reason, the interaction term was excluded from the final model to ensure analytical robustness. Results are presented as adjusted odds ratios (aORs) with 95% confidence intervals.

### Ethical Considerations

Ethical approvals for the primary study [11] were obtained from the Health Research Ethics Committee of the College of Medicine, University of Lagos (approval number: CMUL/HREC/5/24/1464 obtained on 15th May 2024) and Lagos University Teaching Hospital (approval number: ADM/DSCST/HREC/APP/6566 obtained on 10th May 2024). Written informed consent was obtained from each participant before enrollment, with a detailed explanation of the study objectives, confidentiality safeguards, and the rights to voluntary participation. Participants were assured that declining or withdrawing from the study would not affect their access to clinical services. Data confidentiality was strictly maintained through encryption and password-protected databases. The study aligns with the Declaration of Helsinki and Nigeria’s National Code of Health Research Ethics, ensuring the protection of participants’ autonomy, privacy, and welfare.

## Results

A total of 180 women with complete baseline data were included in the analysis. Overall, 93 women reported having undergone cervical cancer screening within the previous 10 years, yielding a screening uptake of 51.7% (95% confidence interval: 44.1–59.2). The mean age of participants was 43.6±7.2 years, and women who had been screened were significantly older than those who had never been screened (45.4±6.7 vs. 41.7±7.2 years; *p*<0.001). Table 1 presents participant characteristics by screening status. No statistically significant differences were observed between screened and unscreened women across key sociodemographic variables, including marital status, educational attainment, employment category, income class, tribe, and residential distance to a screening facility.

After adjusting for age and tribe in a logistic regression model, income class was not found to be independently associated with screening uptake. Compared with women in the low-income category, those in the middle-income (adjusted OR = 1.72, 95% CI: 0.75–3.98) and high-income (adjusted OR = 1.22, 95% CI: 0.45–3.30) categories had higher but statistically nonsignificant odds of screening. An interaction term between education and income level was initially included to examine potential effect modification; however, its inclusion led to model instability, reflected by excessively large standard errors and poor model convergence. For this reason, the interaction term was removed, and the final model was estimated without it [Table 2].

## Discussion

This descriptive cross-sectional analysis of the *mHealth-HPVac* study data assessed the effect of socioeconomic status (SES) on cervical cancer screening uptake among mothers of vaccine-eligible adolescent girls in Lagos, Nigeria. Approximately half (51.7%) of the participants reported having been screened for cervical cancer within the last ten years, indicating a moderate but suboptimal level of screening uptake relative to the national [14] and World Health Organization (WHO) target [15]. This is consistent with reports from similar urban Nigerian settings [16], where cervical cancer screening remains suboptimal despite the known benefits of early detection and prevention, despite the WHO-recommended projection of 70% screening coverage among women aged 35–45 years by 2030 as part of the global strategy to eliminate cervical cancer as a public health problem [15]. The observed coverage in this study underscores the persistent gap between global aspirations and local realities in many low- and middle-income countries (LMICs), including Nigeria.

Consistent with previous studies, age was the only variable significantly associated with screening uptake in our analysis, with older women more likely to have been screened compared to their younger counterparts. This finding aligns with reports from other Nigerian and sub-Saharan African studies showing that screening uptake increases with age [17–19], possibly due to cumulative health encounters, higher risk perception, and increased exposure to reproductive health education [17,20]. Conversely, younger women may underestimate their risk or perceive cervical cancer screening as unnecessary until they experience symptoms, a misconception that contributes to delayed screening.

Contrary to expectations, SES was not independently associated with screening uptake after adjusting for age and tribe. This result diverges from the broader literature, which consistently identifies socioeconomic position as a key determinant of cervical cancer screening uptake in both high- and low-resource settings [21–24]. A plausible explanation may be that, within the urban context of Lagos, socioeconomic disparities exert less influence due to the relatively high concentration of health facilities and targeted awareness programs that have improved access across social strata. Moreover, cultural attitudes, perceived stigma, and fear of diagnosis have been shown to outweigh economic factors in determining health-seeking behaviour for cervical screening in Nigeria [25–28]. This finding may also reflect the availability of subsidised or free screening programs within Lagos, such as those implemented by non-governmental organisations [29], which may have attenuated the influence of economic status on screening participation. It is also possible that self-reported income and occupational status do not fully capture economic empowerment or financial decision-making autonomy among women, particularly in patriarchal household structures common in the study setting. Furthermore, in testing for effect modification, the interaction term between education and income rendered the multivariable model unstable due to multicollinearity and large standard errors, suggesting interdependence between these variables. This statistical instability has been reported in similar population studies where socioeconomic status indicators are highly correlated [30,31]. Future research employing composite socioeconomic indices or principal component analysis may better capture multidimensional socioeconomic effects on screening behaviour.

This study has a few limitations. First, the cross-sectional nature of the analysis and the likely recall bias due to the reliance on self-reported cervical cancer screening history and our inability to verify all screening records could preclude causal inference. Second, analysis of SES relies primarily on education and employment. However, SES is multidimensional and thus exclusion of household assets, housing characteristics, or family economic size, which were not available in the datasets, may limit validity. Third, the sample was hospital-based, limiting generalizability to community settings. Fourth, due to the study’s secondary analytical framework, residual confounding from unmeasured or inadequately captured variables cannot be excluded. Fifth, as the study participants were mothers attending an outpatient clinic at a tertiary hospital, who are likely more health-aware than the general population, there is a high likelihood of unduly inflated screening uptake estimates, reduced SES variability, and limited generalizability to community samples. Finally, while the study is sufficiently powered to assess broad socioeconomic disparities, it may be underpowered to detect modest associations, which could have limited the comprehensiveness of the explanatory model and partly accounted for the lack of statistically significant associations between SES and screening uptake. Larger, adequately powered population-based studies are therefore needed to validate these findings and better delineate the socioeconomic gradients in screening behaviour among Nigerian women. Nonetheless, the study provides valuable insights into the complex interplay between socioeconomic status and cervical cancer screening behaviours in an urban Nigerian population.

## Conclusions

Our study showed a moderate cervical cancer screening uptake in the enrolled cohort of mothers of vaccine-eligible adolescent girls in Lagos, Nigeria. Socioeconomic status was not independently associated with screening uptake. These findings highlight the need to address structural and behavioural barriers that may be more critical to screening uptake than focusing solely on economic or educational disparities. Future studies with larger samples and longitudinal designs could provide further insight into the complex determinants of cervical cancer screening behaviour in Nigerian women.

## Figures and Tables

**Table 1:** Characteristics of enrolled women stratified by their screening uptake (n=180)

Characteristics	Uptake (%)	Non-uptake (%)	*p*-value
**Participants**	93 (51.7)	87 (48.3)	
**Age, years**	45.4 ± 6.7	41.7 ± 7.2	<0.001
**Residential distance to screening facility, km** ^a^	13.0 (7.1–22.5)	10.1 (5.0–22.0)	0.325
**Previous pregnancies**			
One to two	36 (38.7)	24 (27.6)	0.285
Three to four	43 (46.2)	47 (54.0)	
More than four	14 (15.1)	16 (18.4)	
**Marital status**			
Married	74 (79.6)	69 (79.3)	0.966
Not married	19 (20.4)	18 (20.7)	
**Educational level**			
None/Primary	6 (6.5)	12 (13.8)	0.259
Secondary	39 (41.9)	33 (37.9)	
Tertiary	48 (51.6)	42 (48.3)	
**Employment status**			
Unskilled	14 (15.1)	13 (15.0)	0.726
Semi-skilled	55 (59.1)	47 (54.0)	
Skilled/professional	24 (25.8)	27 (31.0)	
**Income class**			
Low	13 (14.0)	19 (21.8)	0.245
Middle	62 (66.7)	48 (55.2)	
High	18 (19.3)	20 (23.0)	
**Tribe**			
Yoruba	29 (31.2)	32 (36.8)	0.157
Igbo	47 (50.5)	32 (36.8)	
Others	17 (18.3)	23 (26.4)	

Abbreviations: IQR, interquartile range; SD, standard deviation.

a Wilcoxon rank sum test.

**Table 2:** Multivariable analyses of effects of socioeconomic status on cervical cancer screening uptake (n=180)

SES Indicators	Number of screened mothers	Adjusted OR (95% CI)
**Income class**		
High	18/38 (47.4%)	1.22 (0.45 – 3.30)
Middle	62/110 (56.4%)	1.72 (0.75 – 3.98)
Low	13/38 (40.6%)	1.00 (reference)
**Age**		
>45 years	39/57 (68.4%)	7.96 (2.28 – 27.78)
35 – 45 years	50/104 (48.1%)	3.61 (1.10 – 11.84)
<35 years	4/19 (21.1%)	1.00 (reference)
**Tribe**		
Yoruba	29/61 (47.5%)	1.78 (0.79 – 4.01)
Igbo	47/79 (59.5%)	1.11 (0.47 – 2.61)
Others	17/40 (42.5%)	1.00 (reference)

Abbreviations: CI, confidence interval; OR, odds ratio; SES, socioeconomic status Inclusion of the Education and Income interaction term rendered the model unstable (large standard errors and poor convergence); hence, the interaction term was excluded from the final model.

## Data Availability

The authors intend to grant access to the datasets used and/or analysed in the study upon reasonable request from the corresponding author (Kehinde S. Okunade).
